# The Effects of Bromelain Supplementation on Clinical Outcomes, Inflammatory and Oxidative Stress Biomarkers in Patients With Traumatic Brain Injury: A Randomized, Double‐Blind Placebo‐Controlled Clinical Trial

**DOI:** 10.1002/fsn3.70162

**Published:** 2026-07-29

**Authors:** Alireza Gheflati, Mohammad Rashid Mayvan, Mojgan Mehri Ardestani, Seyed Mahdi Mirghazanfari, Ebrahim Hazrati, Mohamad Shahab Kalantar, Vahid Hadi, Ali Jafarzadeh Esfehani, Saeid Hadi

**Affiliations:** ^1^ Nutrition and Food Health Research Center AJA University of Medical Sciences Tehran Iran; ^2^ Department of Nutrition Sciences Varastegan Institute for Medical Sciences Mashhad Iran; ^3^ Department of Nutrition, Food Sciences and Clinical Biochemistry, School of Medicine, Social Determinants of Health Research Center Gonabad University of Medical Science Gonabad Iran; ^4^ Department of Persian Medicine, Faculty of Medicine AJA University of Medical Sciences Tehran Iran; ^5^ Department of Physiology and Iranian Medicine, Faculty of Medicine AJA University of Medical Sciences Tehran Iran; ^6^ Departemnt of Anesthesiology and Intensive Care, Medical Faculty AJA University of Medical Sciences Tehran Iran; ^7^ Metabolic Syndrome Research Center Mashhad University of Medical Sciences Mashhad Iran

**Keywords:** bromelains, inflammation, oxidative stress, traumatic brain injury

## Abstract

Traumatic brain injury (TBI) is the leading cause of death from injury. Many studies have shown that cytokines, chemokines, and oxidative stress play a crucial role in the pathophysiology of TBI. One of the types of interventions that are receiving more and more attention is nutritional support and the use of nutritional supplements to reduce inflammation in these patients. The present study was a double‐blind controlled clinical trial, performed on patients with traumatic brain injury. Patients in the intervention group received 600 mg of oral bromelain daily through gavage, while the patients in the control group received a placebo for 14 days. SOFA, APACHE, and NUTRIC scores were evaluated at each time of blood sampling and 28‐ and 60‐day mortality. Also, clinical and biochemical information (CRP, MDA, IL‐6, and TAC), anthropometry, and nutritional information of patients were recorded during the study. There was no significant difference between the two groups in terms of gender, weight, BMI, MAC, APACHE SCORE, NUTRIC SCORE, SOFA SCORE, and energy intake at the beginning of the study (*p* > 0.05). Bromelain supplementation significantly reduced IL‐6 levels (*p* < 0.001), APACHE score (*p* = 0.011), SOFA score (*p* < 0.001), and NUTRIC score (*p* < 0.001). Furthermore, 60‐day mortality was significantly lower in the bromelain group compared to the placebo group (*p* = 0.036). However, there was no significant effect on CRP (*p* = 0.377), MDA (*p* = 0.061), TAC levels (*p* = 0.185). Bromelain supplementation significantly improved IL‐6 levels, APACHE, SOFA, and NUTRIC scores, and reduced 60‐day mortality in patients with traumatic brain injury. However, its effects on CRP, MDA and TAC levels were not statistically significant. These findings suggest that bromelain may serve as a promising adjunctive therapy to reduce inflammation and improve clinical outcomes in critically ill TBI patients. Further studies with larger sample sizes and longer follow‐up periods are needed to confirm these results.

## Graphical abstract

Bromelain supplementation significantly reduced IL‐6 levels (*p* < 0.001), APACHE scores (*p* = 0.011), SOFA scores (*p* < 0.001), and NUTRIC scores (*p* < 0.001). Furthermore, 60‐day mortality was significantly lower in the Bromelain group compared to the Placebo group (*p* = 0.036). However, there was no significant effect on CRP (*p* = 0.377) or MDA levels (*p* = 0.061).

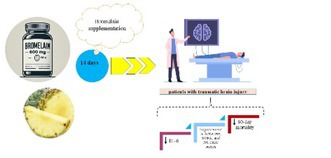

## 1 Introduction

According to the Centers for Disease Control and Prevention (CDC), traumatic brain injury (TBI) is defined as “disruption of normal brain function as a result of several different types of head trauma” (QuickStats C 2010). TBI is one of the common cause of death for people ages 1 to 45, directly responsible for over 50,000 deaths annually in the United States (Langlois et al. 2006; Stocchetti and Maas 2014). Mild, moderate, and severe traumatic brain injuries are clinically categorized using the Glasgow Coma Scale (GCS), with corresponding disability rates of 10%, 60%, and 100% and mortality rates of 20%–30%. An Iranian study by Kavosi found that in 2015, TBI caused by accidents cost over 6.2 billion rials to treat and resulted in over 6390 years of lost life (Kavosi et al. 2015).

A brain injury can cause a hole in the blood–brain barrier, inflammation of the neurons, and ischemic damage, all of which add to the inflammation that is already present in the brain. This issue can lead to poor treatment results or even cell death (Genton and Pichard 2011). The brain's high oxygen requirement and associated production of oxygen metabolites make it both susceptible to and equipped to respond to oxidative stress. Increased production of reactive oxygen species (ROS) is associated with oxidative stress and conditions of severe brain inflammation. Proteins and DNA are two examples of biological macromolecules that may be at risk from these substances. When the production of reactive oxygen species (ROS) exceeds the antioxidant system's capacity (as in the case of a head injury), the recovery process is slowed, the risk of functional defects in the body increases, and the probability of survival decreases (Tayefi‐Nasrabadi and Khodarahmi 2007; Wang et al. 2016).

Inflammatory cytokines, such as IL‐6, cause the transcription of proteins involved in the acute inflammatory phase, such as C‐reactive protein (CRP). Increasing evidence suggests that CRP plays a central role in inflammation, host defense, apoptosis, phagocytosis, and cytokine production (especially IL‐6) (Sproston and Ashworth 2018). Anti‐inflammatory and antioxidant dietary supplements and foods may be helpful for patients in this group in addition to pharmaceutical treatment.

Bromelain is a mixture of proteolytic enzymes derived primarily from the stems and fruit of the pineapple plant ( *Ananas comosus* ). It has been traditionally used for its anti‐inflammatory, analgesic, and digestive properties. Bromelain works by breaking down proteins into smaller peptides and amino acids, which may contribute to its therapeutic effects, particularly in conditions involving inflammation and swelling, such as traumatic brain injury (Roger 2006; Tochi et al. 2008). Bromelain consumption decreases plasma bradykinin, prostaglandin E2, and thromboxane A2, and it increases the anti‐inflammatory cytokine prostacyclin in a dose‐dependent manner (Tochi et al. 2008). In vitro analysis shows that bromelain can reduce serum bradykinin levels and prevent the expression of inflammatory genes like TLR4, TNF‐a, and IL‐8. Cytokines, particularly IL‐8, play a crucial role in attracting granulocytes and monocytes to the location of inflammation, where they exhibit enhanced phagocytic and chemotactic activities (Tochi et al. 2008; Maurer 2001; Rathnavelu et al. 2016; Verma et al. 2017). This provides evidence that bromelain, in conjunction with other analgesics and anti‐inflammatory drugs, may help reduce inflammation in several different disorders (Rathnavelu et al. 2016; Verma et al. 2017).

Since there is no consensus on the effects of bromelain supplementation in TBI patients, the aim of this study was to investigate the effects of bromelain supplementation on clinical outcomes, inflammatory, and oxidative stress biomarkers in TBI patients.

## 2 Materials and Methods

### 2.1 Study Design and Setting

This research is a randomized, double‐blind placebo‐controlled clinical trial. This project was started in May 2023 and finished in October 2023. In this study, 58 patients suffering from traumatic brain injuries admitted to the intensive care unit were selected based on inclusion and exclusion criteria by a skilled neurosurgeon. Fifty‐eight patients who had been diagnosed with epidural hemorrhage (EDH), subdural hemorrhage (SDH), subarachnoid hemorrhage (SAH), brain edema, intracerebral hemorrhage (ICH), or intraventricular hemorrhage (IVH) were selected for this investigation. This study was confirmed in the ethics committee of AJA University of Medical Sciences with the ethics code: IR.AJAUMS.REC.1401.182. This trial was registered at the Iranian Registry of Clinical Trials as IRCT20181219042047N2.

### 2.2 Eligibility Criteria

The inclusion criteria for traumatic brain injury patients were: (a) TBI patients aged 18–65 years old; (b) admission to a Neurological intensive care unit with all types of TBI diagnosis; (c) moderate to severe traumatic brain injury (7 ≤ Glasgow Coma scale ≤ 12); (d) filling out the informed consent by the patient or family members; (e) Maintaining stable hemodynamic and metabolic conditions within the first 24–48 h; (f) having enteral feeding.

The exclusion criteria included: (a) severe and active bleeding; (b) patients treated with inotropic and corticosteroid drugs; (c) Any history of autoimmune disease, cancer, or metabolic disease; (d) pregnancy and lactation; (e) refuse to continue the study; (f) acute or chronic liver failure; (g) chronic renal failure; (h) acute renal failure requiring dialysis; (i) unwillingness to continue participating in the study at any stage of the project implementation; (j) suffering from sepsis or any acute or chronic infection during hospitalization; (k) the death of the patient in the first 72 h; (l) allergy to supplements.

Patients meeting the inclusion criteria were randomized (in quadruple blocks based on a blinded randomization list generated by the online tool for clinical trials: https://www.sealedenvelope.com) in a 1:1 ratio to either bromelain (200 mg three times per day) or placebo groups for 14 days. Patients in the placebo group received capsules identical in appearance to those containing bromelain. The placebo capsules were filled with microcrystalline cellulose, an inert and commonly used excipient in clinical trials, to ensure blinding and avoid any active effects. Patient compliance was monitored throughout the study by counting the remaining capsules during each follow‐up visit and maintaining detailed records. Compliance was calculated as the percentage of the prescribed capsules taken by each participant. Patients with a compliance rate below 80% were excluded from the final analysis to ensure the reliability of the results.

### 2.3 Study Assessment

#### 2.3.1 Clinical Outcome Assessment

Clinical measurement tools such as the Glasgow Coma Scale (GCS), Sequential Organ Failure Assessment (SOFA), Nutrition Risk in the Critically ill (NUTRIC) score, Acute Physiology and Chronic Health Evaluation (APACHE) II score, ICU discharge time, and mechanical ventilator duration were documented by an experienced nutritionist at baseline, Day 7, and Day 14. Additionally, the researchers assessed 28‐ and 60‐day mortality by contacting the patients via phone or accessing their electronic medical records.

Data were collected at three main times: at baseline, on the 7th day of intervention, and on the 14th day of intervention. At baseline, demographic, medical, and social history (living situation, marital status, current occupation) were collected by an expert neurosurgeon.

#### 2.3.2 Anthropometric Assessment

Anthropometric parameters were measured by a well‐trained nutritionist at baseline, Day 7, and Day 14. Body weight was measured using a bed scale (Balas Company) and height was estimated using ulnar length. The mid‐arm circumference was assessed by a nonstretched tape at the midpoint between the tip of the shoulder and elbow. The Body Mass Index was determined by dividing the weight in kilograms by the square of the height in meters. All measurements were taken by a certified nutritionist. The study timeline is presented in Table 1.

**TABLE 1:** General characteristics of patients with traumatic brain injury in intervention and placebo groups.

Variables	Bromelain (*N* = 29)	Control (*N* = 29)	*p* ^a^
Age (year)	40.14 ± 17.87	39.59 ± 17.34	0.545
Gender			
Male	13 (44.8)	16 (55.2)	0.431
Female	16 (55.2)	13 (44.8)	
Weight (kg)	62.17 ± 10.28	63.54 ± 10.08	0.620
BMI (kg/m^2^)	21.69 ± 3.22	22.75 ± 3.87	0.266
MAC (cm)	28.51 ± 4.05	27.46 ± 4.06	0.343
APACHEII score	15.86 ± 4.98	14.41 ± 3.38	0.201
SOFA score	7.10 ± 1.34	6.14 ± 1.50	0.013
NUTRIC score	1.86 ± 1.09	1.86 ± 1.12	1
Energy intake (kcal/day)	429.31 ± 186.37	603.45 ± 313.37	0.013

*Note:* All values are means ± SDs.

Abbreviations: APACHE, acute physiology and chronic health evaluation; BMI, Body Mass Index; MAC, mid‐arm circumference; NUTRIC, nutrition risk in the critically ill; SOFA, sequential organ failure assessment.

^a^ Obtained from an independent *t*‐test.

#### 2.3.3 Laboratory Assessment

10 mL of Venous blood were collected at the beginning, 7th, and 14th days of hospitalization in the early morning following an overnight fast. The serum and plasma were separated using centrifugation at 3000 rpm for 10 min, and then the serum samples were stored at −70°C after centrifugation until the assay was conducted. Finally, serum CRP, IL‐6, TAC, and concentrations were quantified by using commercial ELISA kits. The spectrophotometric method was used to measure serum MDA by using the thiobarbituric acid reactive substance technique.

### 2.4 Power Calculation and Sample Size Estimates

Sample size was determined n1=k×∂12×∂22K×Z1−α2+Z1−β∆2 based on Sistanizad et al. (2021), while type 1 (α) and type 2 errors (B) were assumed as 0.05 and 0.2 (power 80%), respectively. By using a formula, the sample size of 24 participants was determined for each group. Eventually, the number of patients in the study was expanded to 29, representing 20% of the total attrition.

### 2.5 Statistical Analysis

Statistical analysis was performed using statistical software SPSS version 22 (IBM Corporation, Armonk, NY, USA). Shapiro–Wilk test was used to determine whether continuous data were normally distributed. The data were represented as frequency (%) and mean ± SD. In order to compare the baseline and clinical characteristics between groups, the following statistical tests was used: Chi‐square test for qualitative variables and Student's independent *t*‐test for quantities. A paired *t*‐test was used to compare the results before and after the intervention, and ANCOVA was applied to identify differences between the two treatment groups while adjusting for confounding variables. Statistical significance was set as *p* < 0.05.

## 3 Results

**FIGURE 1:**
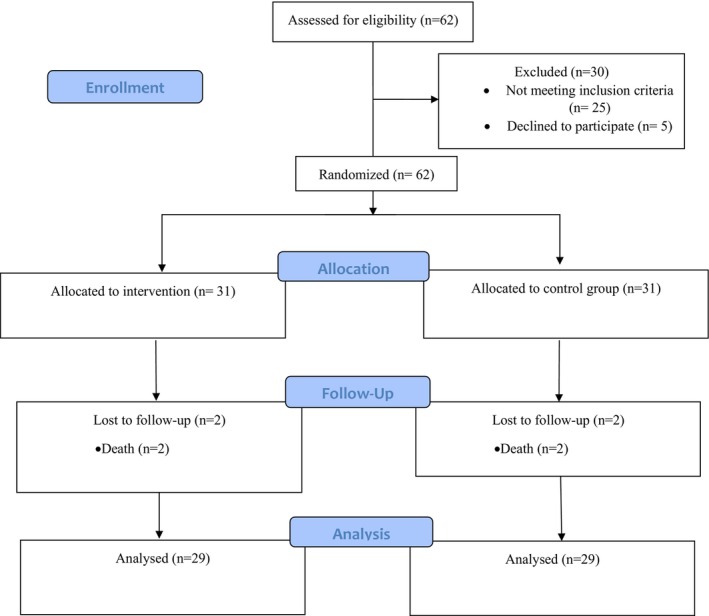
Consort flow diagram of trial.

The recruitment protocol is shown in Figure 1. Three patients in the intervention group were excluded because of death within the first 48 h after hospitalization (*n* = 3). Three patients were also excluded from the control group for death during the first 48 h of hospitalization (*n* = 3). There was no significant difference in the drop‐out rate between groups. Finally, 58 subjects [bromelain (*n* = 29) and placebo (*n* = 29)] completed the trial. No serious side effects were observed in patients with traumatic brain injury who used bromelain throughout the study. However, it should be noted that some participants reported a mild increase in gastric reflux symptoms during the treatment period, which may be attributable to bromelain. The main baseline characteristics of both groups are described in Table 1. No statistically significant differences in the subjects' distribution according to the study's initial parameters. There were no significant differences between the two studied groups in terms of gender, weight, BMI, MAC, APACHE score, NUTRIC score, SOFA score and biochemical parameters at the beginning of the study (*p* > 0.05). At the beginning of the study, no significant differences were observed between study groups in terms of dietary intakes (*p* > 0.05). Also, no difference was found in mean age between bromelain and the control group (40 and 39 years, respectively, *p* = 0.545).

Biochemical outcomes presented in Table 2. By the end of the trial, the bromelain group showed significant improvements in CRP levels (45.08 ± 31.39 vs. 73.44 ± 20.14 mg/L, *p* < 0.001), TAC levels (1610.72 ± 168.60 vs. 1421.44 ± 186.79 μM/L, *p* < 0.001), IL‐6 levels (227.24 ± 66.82 vs. 309.38 ± 47.52 pg/mL, *p* < 0.001), and MDA levels (13.98 ± 3.49 vs. 17.65 ± 4.59 μM/L, *p* < 0.001). In the placebo group, significant reductions were noted for CRP (*p* = 0.005) and MDA (*p* < 0.001), while TAC showed improvement (*p* < 0.001). Between‐group analysis revealed a significant reduction in IL‐6 levels in the bromelain group compared to the placebo group (*p* = 0.001).

**TABLE 2:** Inflammatory and oxidative stress status at baseline and Day 14 in intervention and placebo groups.

Bromelain group (*n* = 29)				Placebo group (*n* = 29)			*p* ^a^
Variables	Baseline	End of trial	*p* ^b^	Baseline	End of trial	*p* ^b^	
CRP (mg/L)	73.44 ± 20.14	45.08 ± 31.39	< 0.001	79.86 ± 21.79	58.53 ± 38.93	0.005	0.377
TAC (μM/L)	1421.44 ± 186.79	1610.72 ± 168.60	< 0.001	1421.17 ± 149.62	1678.75 ± 204.68	< 0.001	0.185
IL‐6 (pg/mL)	309.38 ± 47.52	227.24 ± 66.82	< 0.001	321.76 ± 53.85	294.90 ± 72.85	0.137	0.001
MDA (μM/L)	17.65 ± 4.59	13.98 ± 3.49	< 0.001	15.24 ± 4.59	11.73 ± 2.56	< 0.001	0.061

*Note:* Variables expressed as mean ± SD. *p*‐value < 0.05 at a 95% confidence interval was regarded statistically significant.

^a^ Obtained from analysis of covariance in the adjusted models (adjusted for baseline values and Body Mass Index).

^b^ Paired *t*‐test was used to compare pre‐post tests.

Biochemical parameter data reported in Table 3. The bromelain group demonstrated significant improvements in APACHEII scores (14.14 ± 4.17 vs. 15.86 ± 4.98, *p* = 0.011), SOFA scores (3.76 ± 1.97 vs. 7.10 ± 1.34, *p* < 0.001), and NUTRIC scores (0.93 ± 0.53 vs. 1.86 ± 1.09, *p* < 0.001). In contrast, the placebo group showed no significant changes in APACHEII or NUTRIC scores (*p* > 0.05), while SOFA scores improved slightly (*p* = 0.024). Between‐group analysis indicated that the bromelain group had significantly better outcomes for APACHEII (*p* = 0.005), SOFA (*p* = 0.002), and NUTRIC scores (*p* = 0.010). Mortality rates at 28 days and 60 days were lower in the bromelain group compared to the placebo group, although statistical significance was achieved only for 60‐day mortality (13.8% vs. 37.9%, *p* = 0.036). GCS scores improved in both groups, but no significant between‐group differences were observed.

**TABLE 3:** Comparison of clinical parameters between the two groups.

Bromelain group (*n* = 29)				Placebo group (*n* = 29)			*p* ^a^
Variables	Baseline	End of trial	*p* ^b^	Baseline	End of trial	*p* ^b^	
APACHEII score	15.86 ± 4.98	14.14 ± 4.17	0.011	14.41 ± 3.38	15.69 ± 4.71	0.063	0.005
SOFA score	7.10 ± 1.34	3.76 ± 1.97	< 0.001	6.14 ± 1.50	5.21 ± 1.78	0.024	0.002
NUTRIC score	1.86 ± 1.09	0.93 ± 0.53	< 0.001	1.86 ± 1.12	1.72 ± 1.55	0.667	0.010
GCS	7.62 ± 1.80	9.38 ± 2.14	< 0.001	8.62 ± 2.29	9.90 ± 3.31	0.011	0.566
28‐day mortality (*N* (%))	Yes	9 (31)	3 (10.3)		0.052^c^		
	No	20 (69)	26 (89.7)				
60‐day mortality (*N* (%))	Yes	11 (37.9)	4 (13.8)		0.036^c^		
	No	18 (62.1)	25 (86.2)				

*Note:* Quantitative data are presented as mean ± standard deviation (SD), and qualitative data are demonstrated as frequency and percent. *p*‐value < 0.05 at a 95% confidence interval was regarded statistically significant.

Abbreviations: APACHE, acute physiology and chronic health evaluation; GCS, Glasgow Coma Scale; NUTRIC, nutrition risk in the critically ill; SOFA, sequential organ failure assessment.

^a^ Obtained from analysis of covariance in the adjusted models (adjusted for baseline values).

^b^ Paired *t*‐test was used to compare pre‐post tests.

^c^ *p*‐value is reported based on the analysis of the chi‐square test.

## 4 Discussion

In this study, the effects of bromelain supplementation on inflammatory biomarkers, oxidative stress parameters, and clinical outcomes in patients with traumatic brain injury (TBI) were evaluated. The results showed significant reductions in IL‐6 levels and improvements in APACHE, SOFA, and NUTRIC scores in the bromelain group compared to the placebo group. These findings suggest bromelain's potential as an adjunctive therapeutic agent for reducing inflammation and improving clinical outcomes in TBI patients.

This is consistent with the findings of Chermahini, who reported significant reductions in IL‐6 with bromelain supplementation (Chermahini et al. 2021). Important data regarding the impact of bromelain on the production of cytokines was provided by Hou et al. (Hou et al. 2006) and Huang et al. Also, the anti‐inflammatory properties of bromelain have been documented in rat primary microglial cells (Habashi et al. 2016), human U937 macrophages (Kasemsuk et al. 2018), and RAW 264.7 cells (Lee et al. 2018). Further, These results are in line with previous studies that have reported improvements in functional outcomes with nutritional interventions (Alberda et al. 2009; Barr et al. 2004). In an in vitro study, Onken et al., observed a reduction in TNF‐a expression in inflamed tissues of patients with inflammatory bowel disease treated with bromelain (Onken et al. 2008). Similarly, in another study, Stopper et al. demonstrated that bromelain use in inflammatory bowel disease can reduce the expression of TNF‐α (Stopper et al. 2003). Further, Pothacharoen et al. (2021) found that bromelain has the ability to decrease the production of inflammatory cytokine such as IL‐6 and TNF‐α levels in patients with inflammatory conditions.

The inflammatory response plays a pivotal role in the progression of TBI (Woodcock and Morganti‐Kossmann 2013). After brain trauma, disruption of the blood–brain barrier leads to the activation of inflammatory pathways, recruitment of immune cells, and the release of pro‐inflammatory cytokines such as IL‐6 and TNF‐α (Ziebell and Morganti‐Kossmann 2010). Elevated levels of IL‐6 have been linked to worsened neurological outcomes and prolonged systemic inflammation in TBI patients (Woiciechowsky et al. 2002). Bromelain has demonstrated significant anti‐inflammatory properties by modulating these pathways. One proposed mechanism is bromelain's ability to inhibit NF‐κB signaling, a transcription factor that regulates the expression of multiple pro‐inflammatory cytokines, including IL‐6, TNF‐α, and IL‐1β (Rajan et al. 2022). Suppression of NF‐κB not only reduces the production of inflammatory mediators but also attenuates the activation of microglia and astrocytes, thereby mitigating neuroinflammation (Ageeva et al. 2024). In addition to its impact on cytokine production, bromelain has been shown to enhance the activity of anti‐inflammatory cytokines such as IL‐10 (Pereira et al. 2023). By shifting the balance toward anti‐inflammatory responses, bromelain contributes to resolving inflammation and preventing further neuronal damage (Pereira et al. 2023). Furthermore, bromelain can degrade cell‐surface adhesion molecules, such as ICAM‐1 and VCAM‐1, which are critical for leukocyte adhesion and migration to the site of injury (Gwozdzinski et al. 2023). This may explain the reduction in systemic inflammation observed in the current study.

Oxidative stress is another critical factor in TBI pathophysiology (Khatri et al. 2018). The production of reactive oxygen species (ROS) following trauma exacerbates neuronal damage, disrupts cellular signaling, and impairs mitochondrial function (Beckhauser et al. 2016). Bromelain has demonstrated antioxidant properties through its ability to scavenge free radicals and upregulate endogenous antioxidant enzymes, including glutathione peroxidase (GPx) and superoxide dismutase (SOD) (Agrawal et al. 2022; Kumar et al. 2023). These enzymes play essential roles in neutralizing ROS and maintaining redox homeostasis. In the present study, the significant increase in TAC levels in the bromelain group supports the hypothesis that bromelain enhances the body's antioxidant defenses. Bromelain has been reported to neutralize free radicals and alleviate oxidative stress, safeguarding cells from damage and supporting overall health (Lee et al. 2018; El‐Demerdash et al. 2022). The significant increase in antioxidant status in both groups underscores bromelain's potential antioxidant properties, as previously noted by Yenice et al. (Yenice et al. 2019). Also, Prabakar et al. in an in vitro study, investigated the cytotoxicity and antioxidant properties of bromelain. Similarly, Pekas et al. demonstrated that bromelain, in combination with anthocyanins, significantly increased TAC, aligning with our findings on the positive effects of bromelain on enhancing TAC (Pekas et al. 2020). Their results showed that bromelain extracts are excellent natural antioxidants with low cytotoxicity, offering a promising alternative for managing oxidative stress (Prabakar et al. 2021). However, the lack of a significant between‐group difference in MDA levels suggests that bromelain's effects on lipid peroxidation may be dose‐dependent or influenced by the severity of oxidative damage. In contrast with our results, Kasemsuk et al. reported a significant reduction in plasma MDA levels following 16 weeks of bromelain treatment in patients with mild‐to‐moderate knee osteoarthritis (Kasemsuk et al. 2016).

The observed improvements in APACHE, SOFA, and NUTRIC scores highlight bromelain's potential impact on systemic health and recovery. These results are in line with previous studies that have reported improvements in functional outcomes with nutritional interventions (Alberda et al. 2009; Barr et al. 2004). The APACHE score reflects the acute physiological status of critically ill patients (Zimmerman et al. 2006), while the SOFA score evaluates organ dysfunction (Moreno et al. 2023). Bromelain's ability to reduce inflammation and oxidative stress likely contributed to the observed improvements in these scores. Additionally, the NUTRIC score, which assesses nutritional risk in critically ill patients, was significantly lower in the bromelain group. This suggests that bromelain may improve nutrient utilization or metabolic efficiency, which could be linked to its role in modulating inflammatory and oxidative pathways.

The mortality data further underscore bromelain's potential clinical benefits. While the reduction in 28‐day mortality was not statistically significant, the significant reduction in 60‐day mortality in the bromelain group is noteworthy. This suggests that bromelain may have longer‐term benefits that extend beyond the acute phase of TBI. Previous studies have highlighted bromelain's role in improving wound healing and recovery in various conditions, supporting its potential application in critical care settings.

Despite these promising findings, several limitations must be acknowledged. The relatively short duration of the intervention (14 days) may not have been sufficient to fully capture bromelain's long‐term effects on inflammation and oxidative stress. Additionally, the sample size, though adequate for detecting significant changes in primary outcomes, may limit the generalizability of the results. Variability in patient characteristics, such as the severity of TBI and comorbid conditions, could also influence the observed outcomes.

Future research should aim to address these limitations by incorporating larger sample sizes, longer follow‐up periods, and more detailed analyses of molecular mechanisms. Investigating the dose–response relationship of bromelain and its potential interactions with other therapeutic agents could provide valuable insights into its optimal use in TBI management. Moreover, exploring bromelain's effects on other markers of inflammation and oxidative stress, such as TNF‐α, SOD, and GPx, would further elucidate its mechanisms of action.

## 5 Conclusion

In conclusion, bromelain supplementation demonstrated significant anti‐inflammatory and clinical benefits in TBI patients, as evidenced by reductions in IL‐6 levels and improvements in APACHE, SOFA, and NUTRIC scores. These findings highlight bromelain's potential as an adjunctive therapy for managing inflammation and promoting recovery in critically ill patients. However, further studies are needed to confirm these results, optimize dosing regimens, and explore the long‐term effects of bromelain supplementation in diverse patient populations.

## Author Contributions

**Alireza Gheflati:** investigation (equal), methodology (equal), writing – review and editing (equal). **Mohammad Rashid Mayvan:** visualization (equal), writing – original draft (equal). **Mojgan Mehri Ardestani:** data curation (equal). **Seyed Mahdi Mirghazanfari:** data curation (equal). **Ebrahim Hazrati:** conceptualization (equal). **Mohamad Shahab Kalantar:** investigation (equal), supervision (equal). **Vahid Hadi:** resources (equal), software (equal). **Ali Jafarzadeh Esfehani:** formal analysis (equal), software (equal). **Saeid Hadi:** supervision (equal), writing – review and editing (equal).

## Ethics Statement

This study was confirmed in the ethics committee of AJA University of Medical Sciences with the ethics code: IR.AJAUMS.REC.1401.182.

## Conflicts of Interest

The authors declare no conflicts of interest.

## Data Availability

The data that support the findings of this study are available on request from the corresponding author.
